# Placenta Extracellular Vesicles: Messengers Connecting Maternal and Fetal Systems

**DOI:** 10.3390/biom14080995

**Published:** 2024-08-13

**Authors:** Cheryl S. Rosenfeld

**Affiliations:** 1 Biomedical Sciences, University of Missouri, Columbia, MO 65211, USA; rosenfeldc@missouri.edu; 2 MU Institute for Data Science and Informatics, University of Missouri, Columbia, MO 65211, USA; 3 Department of Genetics Area Program, University of Missouri, Columbia, MO 65211, USA; 4 Department of Thompson Center for Autism and Neurobehavioral Disorders, University of Missouri, Columbia, MO 65211, USA

**Keywords:** trophoblast, neurogenesis, exosomes, neurotransmitters, miRNA, preeclampsia, endocrine disrupting chemicals, DOHaD

## Abstract

The placenta operates during gestation as the primary communication organ between the mother and fetus. It is essential for gas, nutrient exchange, and fetal waste transfer. The placenta also produces a wide range of hormones and other factors that influence maternal physiology, including survival and activity of the corpus luteum of the ovary, but the means whereby the placenta shapes fetal development remain less clear, although the fetal brain is thought to be dependent upon the placenta for factors that play roles in its early differentiation and growth, giving rise to the term “placenta–brain axis”. Placental hormones transit via the maternal and fetal vasculature, but smaller placental molecules require protection from fetal and maternal metabolism. Such biomolecules include small RNA, mRNA, peptides, lipids, and catecholamines that include serotonin and dopamine. These compounds presumably shuttle to maternal and fetal systems via protective extracellular vesicles (EVs). Placental EVs (pEVs) and their components, in particular miRNA (miRs), are known to play important roles in regulating maternal systems, such as immune, cardiovascular, and reproductive functions. A scant amount is known about how pEVs affect fetal cells and tissues. The composition of pEVs can be influenced by gestational diseases. This review will provide critical insight into the roles of pEVs as the intermediary link between maternal and fetal systems, the impact of maternal pathologies on pEV cargo contents, and how an understanding of biomolecular changes within pEVs in health and disease might be utilized to design early diagnostic and mitigation strategies to prevent gestational diseases and later offspring disorders.

## 1. Introduction

The role of the placenta as an intermediary between the maternal and fetal systems is broadly appreciated, but the extent to which the placenta shapes fetal development, distinct from its general provisioning role, remains less clear. In rodents, for example, the fetal brain is thought to be dependent upon the placenta for factors that play direct roles in its early development [1,2]. The tight linkage between the two organs has given rise to the concept of a placenta–brain axis [1,2], a term that encapsulates the close relationship between the two organs. The mechanisms by which the placenta regulates maternal and fetal systems, including the central nervous system, demand further exploration, particularly because of their relevance to longstanding health complications that might originate in utero, i.e., both maternal disorders and fetal diseases with a developmental origin of health and disease (DOHaD) basis. Moreover, sampling the placenta or its biological products at birth or maternal plasma or urine during pregnancy for key biomarkers guiding fetal brain development might pave the way for new avenues in early diagnostic and therapeutic strategies.

The placenta produces a variety of hormones and other factors that shape maternal systems, including those that regulate maternal recognition of pregnancy, such as human chorionic gonadotropin (hCG) in humans, placental lactogens and prolactins in rodents [3], and interferon-t (IFNT) in ruminants [4,5,6,7,8]. While such factors might directly transit via the vasculature system, other smaller molecules, including miRNA- miR, originating from the placenta likely need to be encased in a protective shell to avoid metabolism and permit shuttling to maternal and fetal tissues in physiological concentrations. The placenta might also influence fetal systems through the production of other hormones, factors, and small RNA (miR, snoRNA, and other non-coding RNA).

In the fetal brain, serotonin (5-HT) influences almost all essential neurological processes that span from glial formation to synaptogenesis [9,10,11]. 5-HT presence in the human placenta was first reported in 1965 [12], but it remains unclear whether 5-HT is synthesized there [13,14], even though the machinery for its biosynthesis and catabolism and a capacity for its uptake have been demonstrated in certain human trophoblast (TB) cell types [15,16,17,18,19]. 5-HT immunoreactivity is visible in the ectoplacental cone (EPC) of the developing mouse placenta [20]. An apparent concentration gradient from the parietal trophoblast giant cell (pTGC) through the spongiotrophoblast (spongioTB) has been proposed as a potential route for provisioning the fetus, especially the early brain, with 5-HT [21,22]. The junctional zone, which emerges from the distal end of the EPC between embryonic age-E9-12, is comprised of spongiotrophoblast, glycogen cells, and invasive secondary parietal trophoblast giant cells (pTGC) [23,24,25,26,27,28]. In this regard, there appears to be a progressive switch during brain development from an early placental source of 5-HT, which is vital for proper forebrain development, to a later endogenous source from the fetal hind brain [20,21]. That early placental source has been proposed to be either 5-HT accrued from the mother [14,20,29] or synthesized in the placenta at the expense of maternal tryptophan [16,21,30,31,32]. A primary site of 5-HT accumulation, and also that of a second neurotransmitter, dopamine, is the layer of pTGCs located at the interface between the placenta and maternal decidual tissue [33].

**Figure 1:**
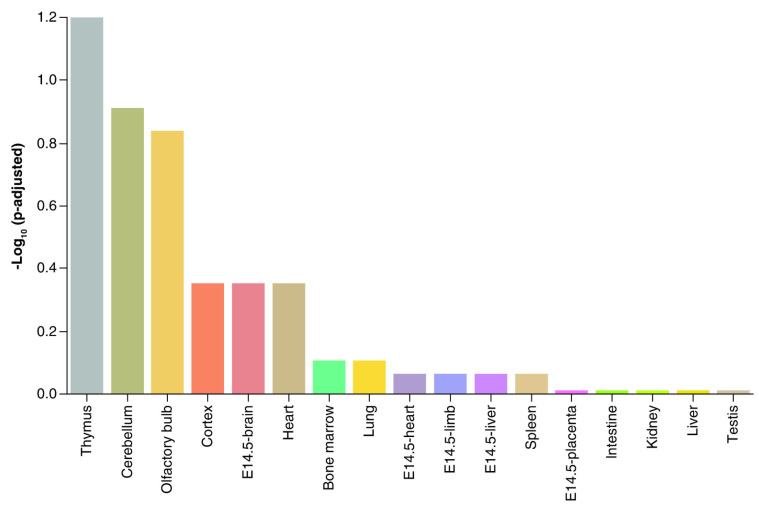
Tissue-specific gene enrichment based on target mRNAs for miRNAs altered in the placenta due to BPA exposure. Tissue-specific gene enrichment analysis based on target mRNAs was determined by TissueEnrich [40]. The mouse ENCODE [41] dataset was used for the enrichment analysis with default settings. Enrichments were considered significant if the *p*-value was ≤0.01 and fold-change ≥2. The target mRNAs were linked to the thymus, cerebellum, olfactory bulb, brain cortex, E 14.5 brain, and heart. E: embryonic age. This figure was published in [34] and reproduced in the current work with permission from Taylor & Francis and Copyright Clearance Center.

We recently reported that developmental exposure to the endocrine-disrupting chemical bisphenol A (BPA) affected placental miRNA profiles [34], while also influencing subsequent offspring behavior [35,36,37,38]. We initially believed that the miRNA whose concentrations became altered by BPA would target mRNAs associated with the placenta. However, we discovered that, instead, the target mRNAs were those likely to be enriched in the thymus and various neural tissues (Figure 1) [34] and especially ones regulating neurogenesis and associated pathways. Another mouse study that examined miR expression patterns in the placenta likewise suggested that placental miRs contribute to fetal brain development [39].

The key question that arises from all these studies is: How do placental catecholamines, miRs, and other small molecules reach the maternal and fetal systems to induce physiological and phenotypic changes? Evidence from our laboratory suggests that it may occur via protective extracellular vesicles (EVs). A vast number of original studies and reviews have explored how placental EVs (pEVs) impact various maternal systems, including cardiovascular, immunological, and reproductive. Thus, we will consider the current state of knowledge on how pEVs impact such maternal systems in health and disease. We will then turn our attention to focus on a topic in its nascent stages, but one that is important from a DOHaD perspective, and that is how pEVs impact the fetal brain and other fetal systems. We will explore how maternal pathophysiological state might impact the cargo contents packaged within pEVs, in particular, miRs, which in turn dramatically influence both maternal and fetal systems. Lastly, we will consider how knowledge of pEV components in health and disease might pave the wave for innovative diagnostic and therapeutic strategies to prevent maternal and offspring diseases with a DOHaD basis.

## 2. Extracellular Vesicles, Cargo Contents, and Critical Tools for EV Analysis

Based on their architecture and contents, EVs are categorized into microvesicles, exosomes, and apoptotic bodies. Exosomes are 30–160 nm in diameter and are released from cells upon fusion of an endocytic compartment, a multivesicular body (MVB), with the plasma membrane [42]. This liberates exosomes into the extracellular milieu [42]. Their outer membranes are protective of their contents and presumably also contain information needed to target the exosomes to remote target organs. EV as a class contain proteins (receptors, transcription factors, extracellular matrix proteins, and enzymes), lipids, DNA, mRNA, miRs, and various metabolites [42].

Several resources are now available to those doing work on EVs. The International Society of Extracellular Vesicles (ISEV) represents the largest scientific organization of researchers exploring the vast range of EVs in health and disease (http://www.isev.org). This organization sponsors one annual meeting, journal clubs throughout the year, and two scientific journals. ExoCarta is an exosome database that provides a rich resource for molecular contents, such as proteins, RNA, and lipids, that have been identified in exosomes across multiple organisms (http://www.exocarta.org). Through this site, investigators can link to FunRich, a standalone bioinformatics tool to analyze EV data; Vesiclepedia, a comprehensive compendium on extracellular vesicles; and external links, including the ISEV organization, Journal of Extracellular Vesicles, and exRNA Research Portal.

**Figure 2:**
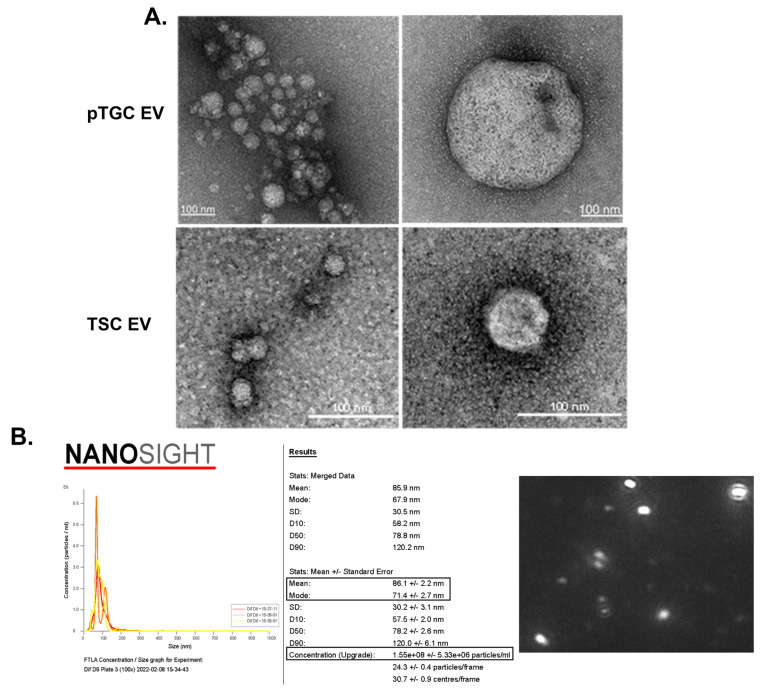
Transmission electron microscopy and NanoSight analysis of pTGCs and TSCs. (**A**) Transmission electron microscopy of EVs derived from pTGCs (top row) and TSC (bottom row). Stained with uranyl acetate. (**B**) Example of a NanoSight report from EVs isolated from culture media containing pTGCs differentiated from TSCs. This analysis provides the average size (mean and mode) of the particles, concentration of particles per ml, and details on Brownian motion based on video analysis of the EVs. A screen capture from the video shows mouse pEVs as viewed with NanoSight. This figure was published in [43] and reproduced in the current work with permission from Oxford University Press and Copyright Clearance Center.

**Figure 3:**
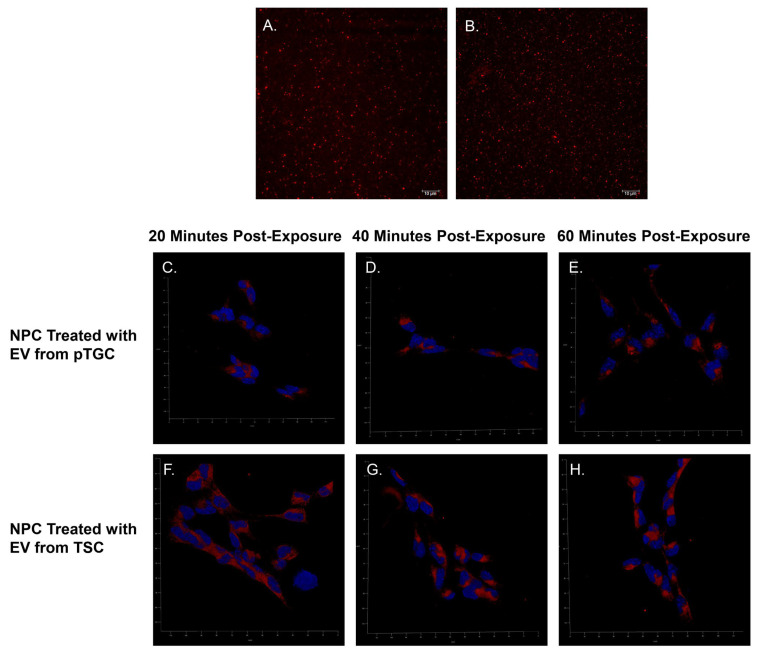
Confocal fluorescent microscopy images of isolated and fluorescently labeled EVs from pTGC and TSC and internalization of TB-derived EV by mouse NPC. (**A**) Isolated and fluorescently labeled EVs from pTGC. (**B**) Isolated and fluorescently labeled EVs from TSC. (**C**–**E**) NPC with EVs derived from pTGC. (**F**–**H**) NPC with EVs derived from TSC. This figure was published in [43] and reproduced in the current work with permission from Oxford University Press and Copyright Clearance Center.

Being only nanometers in size, it is challenging to view and track EVs. Transmission electron microscopy has been the mainstay for visualizing and estimating the size of EVs. However, recent technologies permit additional methods to both visualize and track the movement and potential internalization of EVs from one cell type to another. NanoSight Pro (Malverne Panalytical, Malverne, UK) equipment is essential for EV work, as it provides high-resolution imaging based on light diffraction and quantitation of particles ranging from 10 to 1000 nm in terms of size (mean and mode) and concentration within a liquid media. Example Nanosight data with EVs derived from mouse TB cells are provided in Figure 2, which shows the movement of individual particles [43]. Several companies, including System Biosciences (Palo Alto, CA, USA) and Thermo Fisher Scientific (Waltham, MA, USA), also now offer various kits to fluorescently label EV that aide in both quantification and internalization. For instance, Figure 3 illustrates fluorescently labeled EVs derived from mouse TB and their subsequent internalization by mouse neural progenitor cells [43]. All these methodologies are key in analyzing EVs released into culture media and in vitro-based studies. The critical issue with pEVs will be following their release and delivery to both maternal and fetal tissues. Such in vivo studies have not been conducted. However, by utilizing a recently developed mouse model, detailed below, we are in the process of creating a transgenic mouse model that will permit us to visualize the release of pEVs and their delivery/uptake to maternal and fetal cells. Such pioneering studies will permit us to determine whether EVs are the primary mechanisms by which the placenta, during its short lifespan, communicates and permanently shapes maternal and fetal health.

## 3. Effects of Placental Extracellular Vesicles on Maternal Systems

The majority of studies on pEVs has described how their contents are influenced by the mother and change with gestational diseases (e.g., preeclampsia, PE; and gestational diabetes mellitus, GDM) [44,45,46,47,48,49,50,51,52,53,54,55,56,57,58,59]. Placenta-derived EVs have been proposed to be a “gold mine in translational research and regenerative medicine” [60]. For this section, we will consider how pEVs and biomolecules within such structures impact maternal cardiovascular, immunological, and reproductive functions. Two main types of pEVs in humans have been studied in relation to these systems: those derived from synctiotrophoblasts (SynctioTB), which are in direct contact with maternal blood, and ones originating from extravillous trophoblasts (EVT) that invade the underlying uterine spiral arteries. Thus, where TB-specific EVs have been examined, such information will be provided below. Another approach that has been used to examine how pEVs influence maternal systems is to inject human pEVs into pregnant rodent models and then determine the impacts on maternal or fetal tissues. Such studies are also included below, with the caveat that human-derived forms might act differently in such rodent models, as there are variances in rodent vs. human TB cells and their EV products.

### 3.1. Effects of Placental EV on Maternal Cardiovascular Responses

A considerable number of studies have researched the role of pEVs in regulating normal pregnancies and changes in pEV composition in women with PE. Synctiotrophoblast EV (STBEVs) from normal pregnancies compared to those obtained from PE pregnancies resulted in impaired maternal endothelial function by decreasing nitric oxide bioavailability. While STBEVs from both pregnancies increased NF-κB activation, reactive oxygen species, nitrotyrosine levels, and decreased NO levels by human umbilical vein endothelial cells, those from PE pregnancies led to less robust responses than STBEVs from normal pregnancies [61]. Collectively the findings suggest that the molecular composition of STBEVs can be impacted by PE and may in turn lead to maternal vascular dysfunction. Plasma EVs and pEVs appear to be increased in severe PE compared to normal pregnancies and are linked to various maternal vascular pathologies, including impaired endothelial repair and proliferation, impairments in endothelial cell barrier, reduced endothelial-dependent vasorelaxation, and decreased nitrite levels. Further, pEVs from severe PE caused downregulation of endothelial nitric oxide synthase (e-NOS and p-eNOS) compared to normotensive-derived EVs [62].

Incubation of small EVs released by extravillous trophoblasts (EVTs) subjected to hypoxia with human endothelial cells increased the release of GM-CSF, IL-6, IL-8, and VEGF compared to those not incubated with such structures or normoxic EVs [63]. Injection of hypoxic EV from these cells into pregnant rats increased mean arterial pressure, with elevations in both systolic and diastolic blood pressures. Over 500 proteins within EVs from EVT differed according to oxygen culture conditions (hypoxic or normoxic). Taken together, the findings suggest that oxygen tension can affect the contents within EVs derived from EVT, and such structures might regulate inflammatory and blood pressure responses, thereby contributing to the risk for PE.

Another study suggested that injection of EVs ranging from nano to macro in size from first-trimester human placenta into pregnant mice at day 12.5 resulted by 24 h in anti-constrictive and pro-vasodilatory effects in maternal resistance arteries, implying that EVs might promote maternal vasodilation during pregnancy to increase the blood supply going to the placenta [64]. Isolation of exosomes of presumed placental origin, as indicated by expression of placental-specific marker (PLAP), circulating in the maternal bloodstream from first trimester, second trimester, and third trimester stimulated endothelial cell migration, consistent with the idea that such structures might promote angiogenesis and oxygen delivery to the placenta [65].

EVs derived from porcine trophoectoderm cells stimulated in vitro proliferation of treated maternal endothelial cells, which may promote angiogenesis [66]. Select miRs were enriched in these EVs, with miR-126-5P, miR-296-5P, miR-16, and miR-17-5P being the most abundant angiogenic miRs. Proteins within these structures also appear to regulate angiogenesis. Successful pregnancy hinges on maternal vascular support to the growing conceptus, and thus maternal angiogenesis is likely due to crosstalk occurring via pEVs.

### 3.2. Effects of Placenta EV on Maternal Immune System Responses

Being only 50% related to the mother, there is a great deal of interest in understanding how the placenta might suppress maternal immune responses across a range of species. The miRs and other contents within pEVs have been shown to induce mixed effects on maternal immune cells and pathways, as reviewed below. One of the first studies in this area showed that immunomodulatory proteins, HLA-G5, B7-H1, and B7-H3, were secreted in microparticles and exosomes from early and term placenta, and EVs containing these proteins might modify the maternal immunological environment to facilitate tolerance to the semi-allograft fetus [67].

STBEVs that are released from the placenta may then transit into the maternal circulation and actively bind to monocytes and B lymphocyte, whereupon they induce release of some cytokines (TNFα, MIP-1α, IL-1α, IL-1β, IL-6, IL-8) but suppress IP-10 (associated with type 1 immunity) [68]. Thus, STBEVs may promote maternal immune adaptation to the invading placenta and fetus. An earlier study determined that monocyte activation in normotensive and PE pregnancies might be stimulated by STBEVs released from the human placenta, and higher amounts of STBEVs circulating in the maternal bloodstream in PE pregnancies could underpin extreme maternal inflammatory reaction associated with this disease [69]. Additionally, STBEVs from PE pregnancies have been reported to activate neutrophils and stimulate the formation of fibrous extracellular lattices containing DNA, otherwise called neutrophil extracellular traps (NETs) [70].

When human micro-EVs were in vitro cultured with murine leukocytes in vitro or intravenously injected into pregnant or non-pregnant mice, they localized to macrophages and to a lesser extent T and B lymphocytes within the spleen, liver, and lungs, and significantly more EVs interacted with leukocytes in pregnant vs. nonpregnant mice [71]. While it is unclear from this study what sort of responses occur after EVs associated with these leukocytes, they might stimulate increased maternal immune tolerance.

Micro RNA-30d-5p within placental human exosomes has been reported to induce macrophage polarization into alternatively activated (M2) macrophages, which appears to be caused by targeting histone deacetylase 9 (HDAC9) [72]. This decidua-like macrophage status allowed for increased trophoblast migration and invasion but inhibited endothelial cell tube formation and migration. Macrophages treated with placental-derived exosomes did not exert any effect on T-cell proliferation. An earlier study by this same research group found that human placental-derived exosomes contribute to maternal immune tolerance by reprogramming the genetic machinery within circulating monocytes [73]. Specifically, miR-29a-3p within placental-derived exosomes induced monocyte expression of programmed cell death ligand-1, a surface receptor that inhibits adaptive immune responses, and this miR did so by down-regulation of phosphatase and tensin homolog in monocytes. Another report suggested that intermediate-sized trophoblast EVs contained heat shock protein, mitochondrial (HSPE1), and miR cargo that impacted T regulatory cell proliferation [74].

A recent report indicated that 5′ transfer RNA fragments (tRFs) comprise the majority of small RNA in STBEVs in PE and normotensive pregnancies [75]. The 5′-tRF-Glu-CTC is increased in STBEVs from PE pregnancies and was detectable in maternal plasma. These molecules induced an inflammatory response by tissue macrophages but not circulating monocytes, with a lowering of endothelial nitrous oxide synthase (eNOS) by endothelial cells, and thereby such structures might contribute to pathophysiological changes associated with PE.

The combined data provide convincing evidence that the placenta can act as an immunosuppressive organ through components, including proteins and miR contained within pEVs that act on the maternal immune system to promote tolerance to the semi-allogeneic fetus and allow for increase invasion by the fetal placenta. However, PE might impact the molecular load of these structures and result in excessive maternal immune system response. Additional reports on how miRs contained with pEV influence maternal and fetal responses are detailed below.

### 3.3. Effects of Placental EV on the Uterus and Other Maternal Organs

Synchronized communication between the placenta and uterus is vital for successful survival of the conceptus. Both sides exchange EVs as part of this complex dialogue. In this section, we will focus on how pEVs act upon the endometrium. One of the earliest studies in this area demonstrated that bovine uterine fluids contained both EVs derived from uterine cells and TB cells that contained IFNT, CAPG, and AKR1B1 [76]. The latter up-regulated STAT1, STAT2, MX1, MX2, BST2, and ISG15 transcripts in primary uterine endometrial epithelial cells cultured in the presence of these EVs, suggestive of intercellular communication between TB and uterine cells.

Treatment of receptive endometrial analog RL95-2 cells with EVs derived from a TB analog cell line, JAr, or non-TB EVs revealed that TB-derived EVs simulated endometrial cell protein secretions that were beneficial to conceptus development, attachment, and implantation, but non-TB EVs did not induce the same complement of proteins [77]. By using this same approach, TB-derived EVs caused a rapid and dramatic effect on endometrial transcriptome profiles that supported biological pathway essential conceptus implantation, such as extracellular matrix remodeling and receptor activity, G protein-coupled receptor signaling, and cytokine receptor interactions [78,79]. Moreover, around 9% of the transcripts downregulated in TB-derived EV-treated endometrial cells are likely targeted by miRs within these structures [79]. Thus, TB-derived EVs might act on the endometrium to induce transcriptomic changes and the production of proteins to be secreted into the surrounding milieu that are favorable for conceptus development.

Major Histocompatibility Complex, Class I, E (HLA-E), can be shed by TB cells via TB-derived EVs, and proteins in these structures stimulated IFN-γ and VEGFα by uterine decidual natural killer (dNK) cells via HLA-E [80]. The former inhibited Th17 induction, and the latter induced angiogenesis. Both factors are thus necessary to support successful pregnancies. Suppression of TB-derived EV in mice increased the risk for conceptus reabsorption that was reversed by providing exogenous TB-EV. Human cytoTB EVs upregulated decidual stroma cell transcription and secretion of NF-κB targets, including IL8, but this response was inhibited by treatment of these cells with a soluble form of TNFα receptor [81]. These findings provide further support that TB-derived EVs increased decidual cell release of inflammatory cytokines.

Bovine endometrial epithelial cells (EECs) cultured in the presence of exosomes derived from bovine trophoblast cells pre-treated with progesterone (P4) increased endometrial receptivity factors, including integrin αv, β3, Wnt7a, and MUC, which was likely due to altering the extracellular environment, metabolism, and redox state of the cells [82]. The researchers concluded that exosomes from TB cells might be essential in cattle to increase endometrial receptivity and might thus provide a potential avenue for improving reproductive fecundity.

Besides altering maternal immune and uterine responses, pEVs might also act to affect maternal metabolism to promote fetal growth. One report showed that STBEVs containing apolipoprotein-E stimulated hepatic lipid synthesis in a circadian-dependent manner, and this response might provide additional nutrients to the maturing fetus [83].

### 3.4. Effects of MicroRNAs (miRs) and Other Small RNAs Contain within Placental-Derived Extracellular Vesicles on Maternal Systems

Effects of miRs within pEVs on maternal tissues have been a topic of active interest. Many miRs within human pEVs that can be identified in the maternal plasma are associated with an miR cluster on chromosome 19, C19MC [84]. One of the most prominent within this cluster is miR-517a, which seemingly regulates tumor necrosis factor-mediated signaling. Another miR from this cluster, miR-519c, might serve as a potential biomarker for preterm birth due to endotoxin-producing pathogens [85]. The miR-519d within the C19MC appears to have dual functions in regulating critical trophoblast functions and stimulating proliferation but inhibiting migration, and these miRs can be packaged into EV to shuttle to and affect maternal immune cells to promote immune tolerance during pregnancy [86].

The pEV-derived miR-29a-3p induced monocytes to express programmed cell death ligand-1, which is associated with suppression of the adaptive immune system through down-regulation of phosphatase and tensin homolog [73]. Hypoxic conditions result in human trophoblast cells containing greater amounts of miR-1273d, miR-4492, and miR-4417 that can potentiate PE by targeting HLA-G [87]. A reduction in miR-31-5p within human pEVs has been postulated to be a potential biomarker of PE severity [88]. In contrast, miR-199a-5p was elevated in EVs assumingly secreted by the placenta circulating in the maternal plasma in patients with severe PE, and this miR might hinder endothelial cell function by impacting SIRT1 [89]. Maternal circulation of EV-miR-127-3p during the second trimester was negatively associated with birth weight for gestational age (BWGA) score, whereas positive associations were noted for EV-miR-26b-5p [90].

The miR-1290 was enriched in exosomes derived from the trophoblast HTR8/SVneo cell line. This miR promoted in vitro expression of inflammatory markers (IL-6 and -8) and migration of endometrial epithelial cells, as well increased angiogenesis [91]. By targeting LIM/homeobox protein 6 (LHX6), miR-1290 induced epithelial–mesenchymal transition in the endometrial epithelial cell line HEC-1-A.

Small EVs derived from the TB cell line HTR-8/SVneo contain and transmit miR-25-3p. LPS reduced expression of this miR in TB-derived EVs, and treatment of human myometrial smooth muscle cells (HSMSs) with LPS-primed EVs resulted in enhance expression of Cav3.2 and SERC2a and triggered Ca^2+^ oscillation and contraction of these cells [58]. If such effects were to occur in vitro, premature labor might ensue. Notably, overexpression of miR-25-3p in TB cells reversed these effects in HSMSs treated with EVs derived from the modified TB cells. In pregnant mice, upregulation of miR-25-3p decreased oxidative stress, general inflammatory state, and incidence of premature labor in LPS-treated mice, further evidence that this miR contained with pEVs might help abate myometrial muscle contractions and premature labor.

## 4. Effects of Placental Extracellular Vesicles on the Fetal Brain and Other Fetal Tissues

The fetal brain is especially dependent upon the placenta for nutrients and other growth-promoting factors via the placenta–brain axis [1,2]. Elucidating the crosstalk that occurs between the placenta and brain might provide insight into the origin of neurobehavioral disorders that originate in utero, such as autism spectrum disorder (ASD) As detailed above, the catecholamine serotonin-5-HT is essential for normal fetal brain development [9,10,11]. The placenta might provide an early source of 5-HT for fetal forebrain development. There is likely a progressive switch during brain development from an early placental source of 5-HT, essential for forebrain development, to a later endogenous source from the fetal brain itself [20,21]. That early placental source has been proposed to originate from internalized maternal 5-HT [14,20,29] and/or synthesized by the placenta at the expense of maternal tryptophan [16,21,30,31,32]. Regardless of the source, it is crucial to determine how 5-HT, dopamine, and possibly other catecholamines shuttle from the placenta to the brain without being metabolized such that physiological concentrations can be achieved in this target organ. Other smaller molecules are at risk of metabolism should they have to transit from the placenta to the brain via the fetal vasculature, in particular, miRs.

We discovered that developmental exposure to BPA affected placental miR profiles in mice that target mRNA associated with the thymus and various neural tissues rather than TB cells [34]. Pathway enrichment analyses revealed that the mRNA targeted by placental-associated miRs primarily regulated neurogenesis and associated pathways in the brain. Another report showed similar findings [39]. Ablation of the placental mammal-specific miR379-410 cluster in the mouse resulted in hyper-social behavior, increased excitatory synaptic transmission, and enhanced expression of ionotropic glutamate receptor complexes in the hippocampus [92]. The results indicated that this placenta-specific miR cluster acts to modulate social behaviors and excitatory impulses. A human cohort study demonstrated associations between placental miRs and mRNA and intellectual and social impairments [93], providing further evidence that miRs serve as another key link in the placenta–brain axis. The key question that arises from all these studies is: How do placental catecholamines and miR reach the brain intact to shape gene expression and thereby development of this organ? One possibility is that these placental products are transported to the brain in EVs, where they would be protected from degradation and can transit to distal target organs.

**Figure 4:**
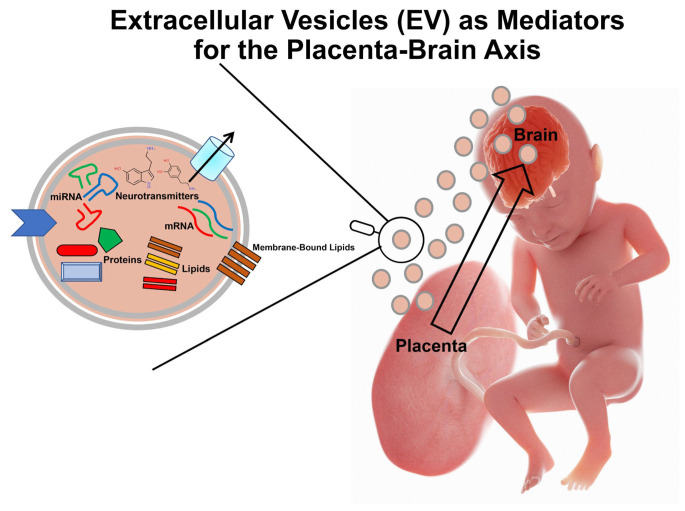
Potential model of how pEVs might function as messengers between the placenta and developing fetal brain. Cargo contents of these structures might include catecholamines, miRs, mRNA, proteins, and lipids, and such biomolecules can thus assumingly transit from placenta to the brain via the fetal vasculature in the protective structures.

To determine whether pEVs might influence the fetal brain and other organs, we analyzed the protein, catecholamine, and small RNA content of EVs from mouse TB stem cells (TSCs) and TSC differentiated into pTGCs, presumably the primary purveyors of 5-HT. We have then analyzed how exposure of mouse neural progenitor cells (NPCs) to EVs from either TSCs or pTGCs affect their transcriptome profiles [43]. The EVs from both TB cells contained physiological concentrations of 5-HT, as well as dopamine and norepinephrine. The miR and small nucleolar (sno)RNA profiles varied based on EV source, with snoRNA exclusively upregulated in EVs from pTGCs. The predicted targets of the miRs from both pTGCs and TSCs were transcripts enriched in the fetal brain. NPCs rapidly internalized EVs, which led to dramatic transcriptomic alterations in neural-associated genes. Transcripts in EV-treated NPCs showed strong linkages with miRs in EVs that were mainly up-regulated and had known associations with neuronal processes. Our results provide robust evidence that pEVs are likely essential for normal fetal brain development and might be integral messengers in the placenta–brain axis (Figure 4) [43].

There are select other reports on how pEVs impact the placenta and fetus. The miR and proteins within pEVs have also been proposed to regulate inflammation and trophoblast invasion [94] and to stimulate acute and chronic inflammation, leading to fetal brain damage [95]. EVs isolated from PE plasma and hypoxic mouse placenta caused disruption of the blood–brain barrier that could render the maternal and emerging fetal brain more susceptible to pathogenic microorganisms [96]. The miRs within pEVs fluctuate throughout pregnancy, and the expression pattern of some miRs correlates with eventual birth weight [93].

Exosome-enriched EVs derived from mouse syncytial TB cells contained a variety of miRs, with Mmu-miR-292-3p and mmu-miR-183-5p being the most prevalent [97]. Predicted mRNA targets for these two miRs are transcripts associated with ubiquitin-mediated proteolysis and intracellular signaling pathways. The miRs contained with these pEVs may thus affect both maternal and fetal systems [97].

The major limitation to date has been being able to visualize the transit of pEVs to their target organs. As such, the work to date has been done with in vitro approaches, including our own study examining the effects of pEVs on NPCs [43]. A recently created mouse model that is commercially available from Jackson Laboratory, termed TIGER knock-in mice (transgenic inducible GFP EV reporter), Gt(ROSA)26Sor^tm1(CAG-CD9/GFP)Dmfel^ that express a Cre-inducible His-tagged CD9/TurboGFP, might be useful for in vivo tracking of pEVs [98]. This transgenic mouse model relies on the abundant expression of CD9 in EVs. Upon breeding males to female mice containing Cre recombinase, the lox-stop-lox cassette is excised such that only Cre+ cells have labeling of their EV with CD9-GFP, resulting in the ability to visualize via intrinsic fluorescence or by means of its polyhistidine-epitope tag and EV trafficking to other organs. To label TB cells of interest, namely, pTGCs and SpaTGC, we are in the process of pairing the TIGER knock-in mice to *Prl2c2*^Cre/-^ mice [99]. This transgenic knock-in mouse model will presumably allow us to track the shuttling of pEVs to fetal organs and possibly even maternal systems. It is also clear that maternal state can affect the composition of pEVs, and thus, we will consider how maternal diseases impact these structures.

## 5. Effects of Maternal Diseases on Placental Extracellular Vesicles

Maternal physiological and disease state can greatly influence the contents of pEVs and thereby their impact on maternal and fetal systems. The studies detailed above have already touched on the fact that PE can induce profound effects on miRs and other biomolecules within pEVs. Additional studies in this area will be reviewed in this section. Further, we will consider how communicable and other non-communicable maternal diseases affect pEVs.

### 5.1. Maternal Non-Communicable Diseases and Effects on Placental Extracellular Vesicles

Placenta accreta spectrum (PAS) is a condition typified by excessive placental invasion into the underlying uterine tissue that increases the risk of obstetric hemorrhage and other adverse maternal and neonatal outcomes. No biomarkers exist for this gestational disorder. A cohort study that analyzed STBEVs within plasma of with women with placenta previa (PP), which increases the risk for PAS-associated disorders, vs. pregnant women without this condition, determined that the former had significantly elevated levels of plasma STBEVs, suggestive that STBEV concentrations might be a barometer for women at risk for this disease [100].

PE is the main maternal disease linked with changes in the composition of pEVs, some of which have already been discussed above. We will consider additional ones below that highlight two aspects of this emerging field: (1) the possibility that pEVs contribute to the pathophysiology of PE and other maternal diseases and (2) the potential to diagnose and prevent this gestational order based on changes in miRs, protein, and other biomolecules contained within pEVs. Several proteins linked to innate immune responses, such as complement and TLR signaling, and hemostasis and oxygen homeostasis have been found to be increased in pEVs from PE pregnancies [101]. Placental exosomes levels for miR-520-5p were reduced in pregnancies where either severe PE or fetal growth restriction was diagnosed [102]. The miR-9-5p was elevated in STBEVs and circulating EVs from pregnancies with PE, highlighting this potential miR as a candidate biomarker for PE [103]. Placental-derived exosomes from PE patients caused endothelial barrier breakdown due to reduced expression of VE-cadherin in endothelial cells [104]. Elevated expression of miR-125b in PE placenta exosomes suppressed VE-cadherin by human umbilical vein endothelial cells, suggesting that this miR modulates the adverse effects of PE placenta exosomes on endothelial barrier function.

In vitro assessments of the effects of STBEVs from PE and normal pregnancies on vascular contractility of human subcutaneous arteries revealed that the former induced more pronounced angiotensin-II-mediated contractions and structural disturbances [105]. Notably, the harmful effects of STBEVs from PE pregnancies could be blocked by inhibiting vesicle uptake by endothelial cells via treatment with chlorpromazine or antibodies directed against LOX-1 receptor. These studies warrant further exploration of the therapeutic potential of blocking vesicle internalization to mitigate cardiovascular damage. Melatonin has also been suggested to be useful in reducing the amount of misfolded proteins within pEVs from PE pregnancies and decreasing the activational ability of these structures on endothelial cells [56].

Most of the reported studies for changes in pEVs secondary to PE report on how these structures affect maternal systems, but one report suggests that these pEVs might also lead to adverse outcomes on the fetus, namely, kidney development. In vitro studies revealed that placental-derived exosomes from PE decreased glomerular endothelial cell proliferation, tubular formation, migration, and barrier functions [106]. Ex vivo studies confirmed that such structures inhibited the growth and branch formation of fetal kidney explants and decreased expression of VE-cadherin and occlusion. Treatment of pregnant mouse placenta with exosomes from PE resulted in fetal renal dysplasia, reduced glomerular number, and decreased filtration barrier. Thus, by altering the molecular cargo within pEVs, PE can lead to permanent offspring health effects.

Analyses of pEVs from pregnant women with multiple sclerosis (MS) vs. healthy pregnant women showed that pEVs from both groups were enriched with surface markers associated with stem/progenitor cells [107]. Treatment of monocytes with pEVs from both groups revealed that those cultured in the presence of pEVs from women with MS had decreased expression of pro-inflammatory cytokines, and decreased proliferation of Treg cells occurred in cells treated with these same pEVs. Thus, the immunomodulatory properties of pEVs from women with MS appear to be compromised.

GDM is a common metabolic disorder experienced by pregnant women who have insulin resistance and decreased circulating glucagon-like peptide-1 (GLP-1) that regulates glucose-dependent insulin secretion. Delivery of the conceptus resolves this condition, thereby suggesting that the placenta might mediate this gestational disorder. STBEVs were increased in women with GDM. STBEVs from GDM also contained significantly greater amounts of dipeptidyl peptidase IV (DPPIV), which increases the breakdown of GLP-1, resulting in disruption of normal glucose homeostatic mechanisms [108]. The results provide compelling evidence that molecular components within STBEVs, in particular, DPPIV, regulate maternal insulin secretion and risk for GDM. A recent study segregated the protein content in small vs. medium/large STBEVs from women with GDM vs. control pregnancies [109]. The protein content in the medium and large STBEVs showed the greatest differences in protein profiles between these two groups, and the overall protein differences might overall impact cytoskeletal organization.

Pregnant women with and without GDM revealed those with this disease had reductions in miR 516 5p, miR 517 3p, miR 518 5p, miR 222 3p, and miR 16 5p within placental exosomes excreted in the urine [110]. Another study determined that placental exosomes isolated from women with GDM contain miRs associated with skeletal muscle insulin sensitivity [111]. Placental exosomes isolated from GDM pregnancies reduced insulin-mediated migration and glucose uptake in primary skeletal muscles cells isolated from patients with normal insulin sensitivity. Placental exosomes secreted in GDM pregnancies contained more miR-320b, and this miR was found to impair insulin activity, as determined by persistent elevated glucose concentrations obtained in glucose tolerance testing [112]. Concentrations of placental exosomes were significantly increased in GDM pregnancies, and these structures increased the release of proinflammatory cytokines by endothelial cells [113].

### 5.2. Maternal Infectious Diseases and Effects on Placental Extracellular Vesicles

A handful of maternal infectious diseases have been linked with changes in the composition of EVs. One study reported that HIV-positive pregnant women had higher concentrations of total and TB microparticles, and microparticles from these women had elevated expression of MHCII but lower expression of MCP1 [114]. This same report determined that mothers with placental malaria had overexpression of miR-517c. Investigators concluded from these results that such structures might have an immunogenic role and could be used as biomarkers for maternal disease state and adverse pregnancy outcomes.

Rhesus macaque TSC and those differentiated into synctioTB were permissive for infection with Zika (ZIKV) virus strain DAK AR 41,524 [115]. Cells infected with ZIKV showed altered expression of immune-related genes, especially those mediating type I and type III interferon responses. ZIKV exposure of both TB cell types changed their EV cargo contents for mRNAs, miRs, and proteins, including ZIKV proteins, providing further evidence that TB-derived EVs might act as biomarkers for maternal disease state.

EVs from human and bovine TB cells have been shown to contain syncytins, which are endogenous retroviral envelope proteins that induce the fusion of cellular membranes. EVs from placental villous explant culture were found to harbor endogenous retrovirus group W member 1 envelope (ERVW-1, synctin-1) [116], which stimulated the formation of synctioTB via cell fusion [117]. Recently, bovine pEVs were shown to contain bovine endogenous retroviral envelope protein K1 (BERV-K1), which might act as participants in fetal–maternal communication [118].

Trophoblast-derived EVs have also been postulated to exert antiviral activity that can be transferred to recipient cells that internalize these structures. One of the earliest reports in this field showed that cultured human placenta TB cells were highly resistant to viral infection, and this resistance is conferred via exosome-mediated delivery of miRs, namely, those on the chromosome 19 miR cluster that are abundantly expressed in the human placenta [119]. Recipient cells that internalized TB-derived exosomes had attenuation of viral replication by induction of autophagy. TB exosomes containing C19MC miRNA attenuated infection of an osteosarcoma cell line by ZIKV [120]. TB-derived exosomes exhibited the greatest degree of antiviral activity relative to microvesicles and apoptotic blebs and contained a distinct protein and phospholipid profile that might contribute to their antiviral properties [121].

## 6. Usage of Placental Extracellular Vesicles for Early Diagnostic and Therapeutic Strategies to Mitigate Offspring Diseases

Placental-derived EVs are detectable in maternal blood and urine samples and may thus serve as a liquid biopsy for maternal diseases, as well as a risk for later offspring diseases. The studies listed above characterizing the molecular contents of pEVs relative to pregnancy disorders isolated these structures from one of these maternal biological samples. The miR, protein, or biomolecular differences based on pEVs relative to maternal condition might be used to diagnose maternal disease state, such as PE, GDM, placenta previa, or infections, as detailed above. A commonality of the reports above also suggests that biomolecular changes within pEVs might contribute to pathological changes, such as vascular abnormalities detected with PE. Thus, mitigation strategies might be designed to reverse miR or protein changes within pEVs, such as through engineered EVs, or to block uptake of miRs by target tissues. For instance, chlorpromazine, antibodies directed against LOX-1, and melatonin have all been suggested to block vesicle internalization by endothelial cells and adverse effects of these structures [56,105].

Placenta EVs contain specific biological markers that permit segregation from other EVs circulating in maternal blood or in urinary samples. For instance, PLAP is expressed on the surface of pEVs only. By using antibodies against biomarkers, investigators were able to identify miRs in PLAP+ EVs purified from plasma from women diagnosed with placenta percreta (where placental trophoblastic cells abnormally invade the uterus) vs. those diagnosed with placenta previa [122]. Other example reports linking specific pEV changes and maternal diseases are provided below.

The pEV miR-31-5p has been suggested to be a sensitive biomarker and target to alleviate PE [88]. Other miRs within placental exosomes that have been proposed as good predictors for PE include hsa-miR-486-1-5p and hsa-miR-486-2-5p [123]. The STBEVs in PE pregnancies but not control pregnancies were observed to express human leukocyte antigen (HLA)-DR molecules that might be a good predictor of this disorder [57]. Increased expression of synctin-1 and NEP (neprilysin, an abundant membrane-bound metalloprotease, that binds and cleaves a variety of peptides, including vasodilators and natriuretics) within STBEVs might be other specific biomarkers and targets for PE [124,125].

Most of the studies implicating pEVs as diagnostic biomarkers and targets to prevent disease have focused on maternal diseases, in particular, PE. However, the composition of pEVs might also serve as predictors for later offspring disease risk, including ASD and other neurobehavioral diseases. Our in vitro studies implicate mouse pEVs in being able to alter transcriptomic profiles in mouse NPC [43]. If such is the case in vitro, then presumably early diagnosis based on changes in molecules contained within pEVs might be possible, especially as pEVs circulate in maternal blood and can be isolated in the urine of pregnant mothers. By determining the miR, protein, or other compounds that have altered expression in pEVs in gestational diseases or linking such biomolecular alterations with later offspring disorders, new avenues are opened for preventative and remediation strategies through engineered EVs, where the cargo contents are altered to reflect the non-disease state and/or can be loaded to contain therapeutic agents. Engineered EVs have been proposed to have multifunctional applications in treating a variety of diseases, including cancer, retinal disorders, neurodegenerative disease, infectious diseases, osteoporosis, inflammatory bowel disease, and wounds [40,126].

## 7. Conclusions and Future Directions

While a transient organ, the placenta has long been ascribed as being the primary communicating organ between the mother and fetus. The past decades have focused on the role of placenta hormones and other factors, such as hCG in humans and non-human primates and IFNT in ruminants in regulating maternal reproduction and promoting survival of the ovarian CL for the maintenance of pregnancy. A surging number of studies this past decade has revealed that the content of pEVs, including miRs and proteins, might be the primary means by which the placenta influences maternal systems, including reproduction, cardiovascular, and metabolic processes. It is also clear that maternal physiological and disease state can alter the composition of pEVs, in particular, miRs. Such maternal diseases include non-communicable diseases, such as PE, GDM, and myasthenia gravis, and infectious diseases. Thus, a greater understanding of the pEV content in health and disease might be leveraged to develop early diagnostic and mitigation approaches for reversing gestational diseases that impact the mother and her fetus.

Scant amount is known about how the molecular cargo contents of pEVs under varying maternal conditions influence fetal organ development and later risk for diseases, including ASD. Our own studies have shown that pEVs can alter the transcriptome profile of NPC in mice [43]. Similar affects are likely to occur in human NPC, and pEVs might regulate the development of other fetal cells and tissues. Future studies are needed to assess how changes in pEV content, such as miRs, affects fetal programming and DOHaD-based diseases. Moreover, potential sex differences in pEV composition should be considered in relation to health and risk for diseases that demonstrate pronounced sexual dimorphism, e.g., ASD, where boys are at a 3:1 risk for this disease relative to girls [41].

The usage of TIGER knock-in mice [98] that allow for vivo tracking will be essential in understanding the contributory role of pEVs in shaping fetal brain and other organ development under controlled conditions and how maternal diseases and exposure to environmental chemicals such as EDC affect this communication. Development of animal models that lack the ability to produce pEVs or the ability of target cells to internalize these structures will help elucidate how biomolecules contained with pEVs impact the fetal brain and other developing organs. Such animal models will also be invaluable in determining how well the in vitro human and rodent TB cell line recapitulates the normal in vivo physiological and pathological responses.

In summary, the ease of isolating such structures from maternal blood and urine will greatly facilitate our understanding how pEVs impact both maternal and fetal systems and will assumingly lead to improved early diagnostic and remediation strategies to combat maternal diseases that include PE, GDM, and other gestational disorders, and later, offspring diseases with a DOHaD-based origin. For all these reasons, pEVs are predicted in the coming decades to live up to their categorization as a “gold mine in translational research and regenerative medicine” [60].
